# Multi‐omics analysis reveals the regulation of SIRT6 on protein processing of endoplasmic reticulum to alleviate oxidative stress in endothelial cells

**DOI:** 10.1002/ctm2.1039

**Published:** 2022-08-29

**Authors:** Peng Li, Zhenyang Guo, Runyang Feng, Na Wu, Xin Zhong, Zheyan Fang, Yiqing Hu, Xueting Yu, Shuang Zhao, Gang Zhao, Yue He, Hua Li, Junbo Ge

**Affiliations:** ^1^ Department of Cardiology, Zhongshan Hospital, Shanghai Institute of Cardiovascular Diseases Fudan University Shanghai China; ^2^ Department of Cardiology Shanghai Xuhui District Central Hospital Shanghai China; ^3^ Department of Medical Examination Shanghai Xuhui District Central Hospital Shanghai China; ^4^ Key Laboratory of Viral Heart Diseases National Health Commission Shanghai China; ^5^ Key Laboratory of Viral Heart Diseases Chinese Academy of Medical Sciences Shanghai China; ^6^ National Clinical Research Center for Interventional Medicine Shanghai China; ^7^ Institutes of Biomedical Sciences Fudan University Shanghai China

To the Editor:

Endoplasmic reticulum (ER) is the largest organelle in cells. ER stress is caused by the protein homeostasis imbalance in ER and contributes to cell dysfunction.^1^ SIRT6, a member of sirtuins, participates in DNA repair, metabolism, inflammation and oxidative stress.^2^ However, it is still unclear whether SIRT6 regulates ER stress in endothelial cells (ECs). In the study, multi‐omics approach was used to reveal a novel mechanism by which SIRT6 alleviated ER stress through maintaining the ER protein homeostasis.

In view of the important roles of SIRT6 in various cellular functions, human microvascular endothelial cells (HMECs) were genetically engineered to construct four cell lines: SIRT6‐kd, SIRT6‐kdnc, SIRT6‐oe and SIRT6‐oenc ECs to explore physiological functions of SIRT6 (Figure S1A). Total 19 835 transcripts, 7120 proteins and 779 metabolites were identified through analysis of transcriptomics, proteomics and metabolomics (Figure 1A–C, Tables S1–3S). The consistency among biological repeats and the divergences among different groups in transcriptomes, proteomes and metabolomes were verified (Figures S1B and S2). The differential genes (fold‐change [FC] >2 or <0.5 and *p‐*value <0.05), proteins (FC >1.2 or <0.8333 and *p*‐value <0.05) and metabolites (FC >1.5 or <0.67 and *p*‐value <0.05) were screened (Figure S1C,D). The kd/kdnc up (upregulated) and oe/oenc down (downregulated) genes were combined into SIRT6 downregulated genes (SDGs), and kd/kdnc down and oe/oenc up genes were combined into SIRT6 upregulated genes (SUGs). Similarly, the SIRT6 downregulated proteins (SDPs) and SIRT6 upregulated proteins (SUPs) were obtained with a total of 311 SDGs, 151 SUGs, 529 SDPs and 330 SUPs (Figure 1C). Further, the SDPs, SUPs, SDGs and SUGs were analysed by KEGG enrichment. The results showed that the pathways of ‘protein export’, ‘protein processing in endoplasmic reticulum’ and ‘ubiquitin‐mediated proteolysis’ were dominantly enriched (Figure 1D). These data implied that SIRT6 might be involved in protein synthesis and degradation in ER.

**FIGURE 1:**
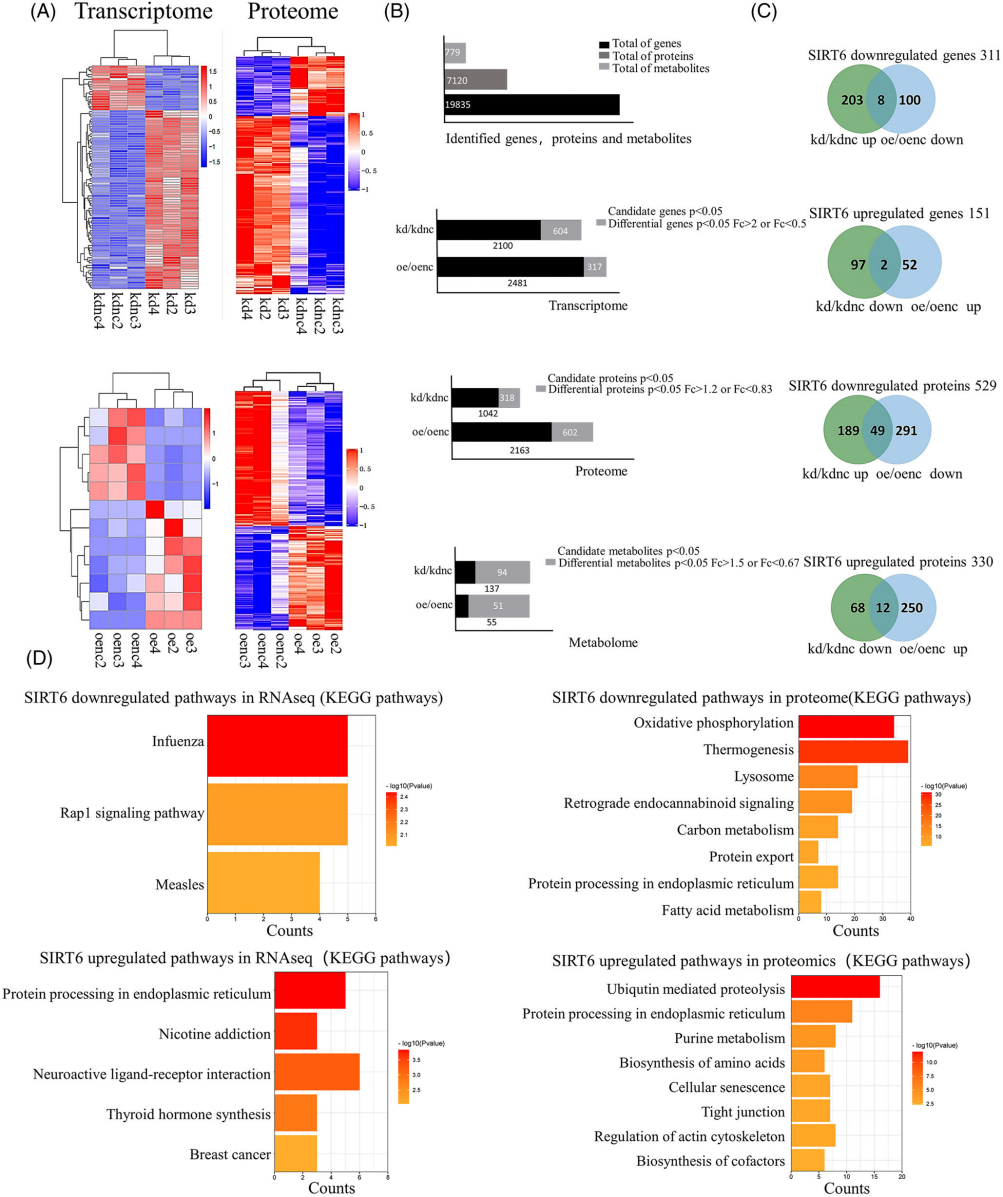
Transcriptomic and proteomic analysis upon SIRT6 knockdown or overexpression. (A) Heatmap of the transcriptome (left) and proteome (right) data. Pearson correlation coefficients were performed to measure the distance and average of sample clustering. (B) Bar plots represent the total number of identified genes, proteins and metabolites (top) and the proportion of differential genes, proteins and metabolites (bottom) in candidate genes, proteins and metabolites with *p* < 0.05 in kd/kdnc group and oe/oenc group. (C) Venn's diagram showing the genes (top) and proteins (bottom) upregulated and downregulated by SIRT6. (D) KEGG enrichment analysis of SIRT6 downregulated 311 genes and upregulated 151 genes (left), and SIRT6 downregulated 529 proteins and upregulated 330 proteins (right). Data are represented as negative of log 10 of *p*‐value after Bonferroni correction.

Through comparing mRNA and protein data, a positive correlation between transcriptome and proteome changes was found (Figure S3). Based on it, the genes and proteins upregulated by SIRT6 and those downregulated by SIRT6 were intersected to obtain 20 strictly differential proteins (Figure 2A). Co‐incidentally, the ‘protein processing in endoplasmic reticulum’ was enriched by the above 20 proteins (Figure 2B). Furthermore, the metabolic footprint of SIRT6 in regulating ER processing was analysed, and 145 differential metabolites were obtained (by FC <0.67 or >1.5 and *p*‐value <0.05). And the differential metabolites pathway enrichment analysis showed that SIRT6 upregulated arginine biosynthesis and sphingolipid metabolism (Figure 2C). In differential metabolites, about 50% of the upregulated metabolites were lipids (Figure 2D). In lipids or sphingolipids, sphingomyelin and its precursor (dihydroceramide and ceramide) were upregulated by SIRT6 (Figure 2E). In addition, aspartate, arginine, glutamate and *N*‐acetyl‐L‐glutamate in urea cycle were also found to be upregulated by SIRT6. These data pointed to the possibility that SIRT6 upregulated arginine biosynthesis to promote urea cycle and protein degradation. About 50% metabolites downregulated by SIRT6 were amino acids, the main material for protein synthesis, which implied that SIRT6 downregulated protein synthesis (Figure 2D). Overall, the results showed that SIRT6 might regulate protein processing in ER by affecting protein transport, protein folding and ubiquitin‐proteasome system (UPS)‐dependent degradation.

**FIGURE 2:**
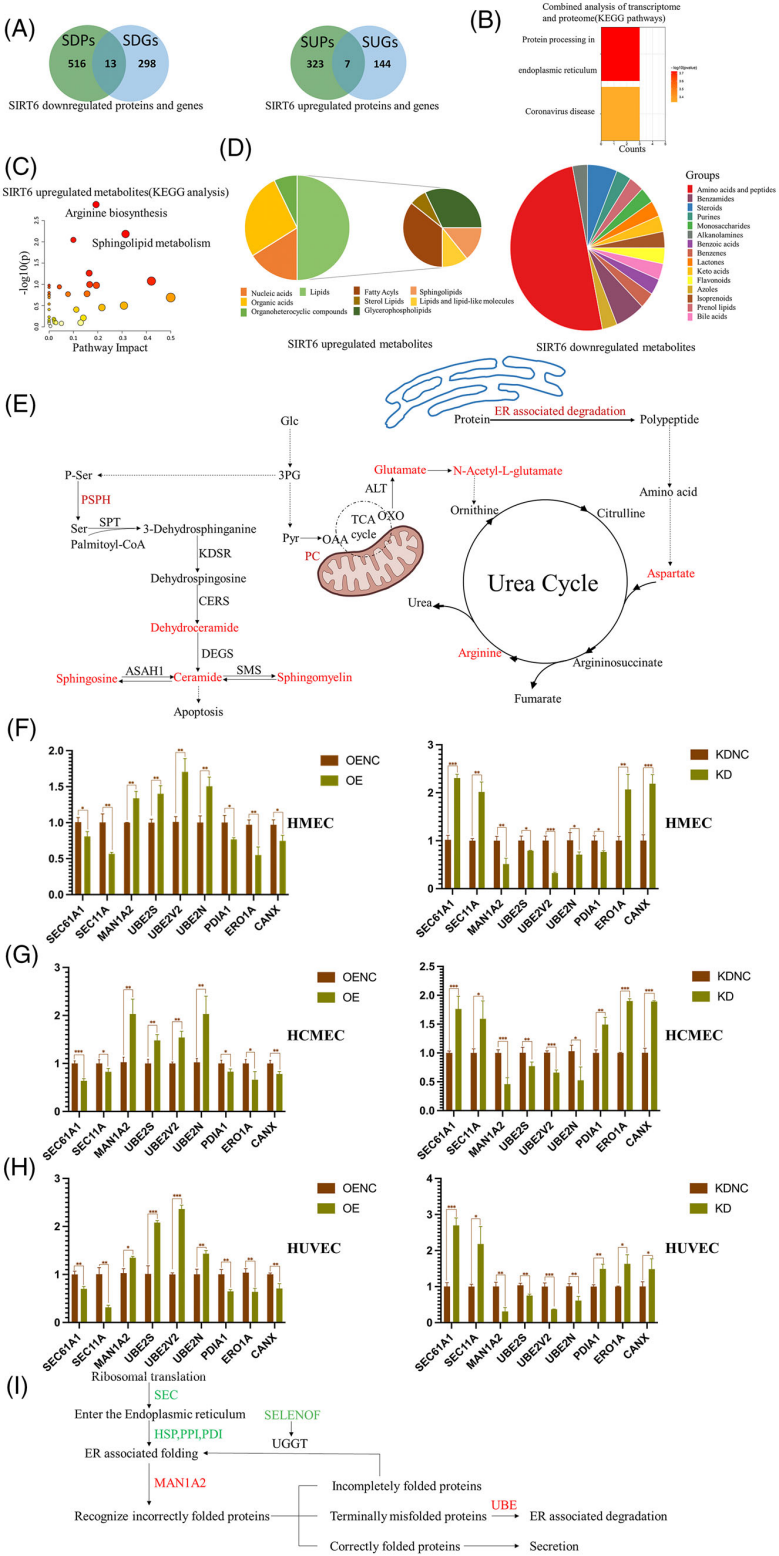
Multi‐omics revealed SIRT6 regulating protein process in endoplasmic reticulum (ER). (A) Venn's diagram showing the strict SIRT6 downregulated (left, both mRNA and protein were downregulated) or upregulated (right, both mRNA and protein were upregulated) proteins by SIRT6. (B) KEGG enrichment analysis of 20 strict differential proteins regulated by SIRT6; data represented as negative of log 10 of *p‐*value after Bonferroni correction. (C) KEGG enrichment analysis of SIRT6 upregulated metabolites. Scatterplots represent *p*‐value from integrated enrichment analysis and impact values from pathway topology analysis using 91 metabolites upregulated by SIRT6. The node colour is based on the *p*‐values and the node radius represents the pathway impact values. (D) The pie chart showing metabolites upregulated (left) and downregulated (right) by SIRT6. (E) Graphical representation of enzymes and metabolites of the sphingolipid synthesis, TCA cycle and urea cycle. Enzymes and metabolites highlighted in red are upregulated by SIRT6, whereas those in blue are downregulated by SIRT6. (F–H) The mRNA levels of key proteins in protein processing in ER pathway in HMECs (F) and HCMECs (G) and HUVECs (H) under oxidative stress. (I) The simple flowchart of protein processing in ER. The new proteins entered the ER by SEC after translation and were folded by HSP, PPI and PDI. Correct folded proteins are sent to secretion. The misfolded proteins were recognised by MAN1A2 and sent to degradation. As for those recognised by UGGT, which is activated by SENLENOF, they will undergo misfolded proteins cycle. Green labelled proteins were downregulated by SIRT6, red labelled proteins were upregulated by SIRT6 in proteomics.

In ER, SEC families and OSTc could transport proteins into ER, and PPI family, PDI family and ERO1a assist in protein folding. Besides them, CANX, CALR, UGGT and SELENOF lead misfolded proteins to enter processing cycle^3^ (Figure 2I). In this study, proteomic data showed that several important proteins were regulated by SIRT6 in above protein families (Figure S4). Subsequently, qPCR was performed to certify whether SIRT6 regulated these key proteins expression. Consistent with the proteomic data, SEC61, SEC11a, PDIA1, ERO1a and CANX were downregulated by SITR6 under oxidative stress in HMECs, human cerebral microvascular endothelial cells (HCMECs) and primary human umbilical endothelial cells (HUVECs) (Figure 2F–H). In addition, proteomic data demonstrated that HSP family proteins such as GRP94 (HSP90B1), ubiquitin binding enzymes (UBE) and ER MAN1 (MAN1A2) were upregulated by SIRT6 (Figure S4). Among them, ER MAN1 stops protein folding cycle, and HSP family promotes the degradation of misfolded proteins, and UBE proteins are indispensable link for degradation^3^ (Figure 2I). By qPCR, MAN1A2 and UBE2S, UBE2V, UBE2N in E2 family were significantly upregulated by SIRT6 under oxidative stress in the above three ECs (Figure 2F–H). These results indicated that SIRT6 could reduce protein entering into ER, decrease the ER‐associated protein folding (ERAF) and promote ER‐associated protein degradation (ERAD), thereby alleviating the protein load in ER.

Considering SIRT6 maintaining ER homeostasis, it is necessary to investigate whether SIRT6 could alleviate ER stress under oxidative stress. The result showed that SIRT6 significantly decreased the reactive oxygen species (ROS) and intracellular Ca^2+^ levels in primary HUVECs treated with H_2_O_2_ (400 µM) (Figure 3A–C). Moreover, the mRNA levels of ER stress markers like ATF6, BIP and CHOP were reduced by SIRT6 in these three ECs. The protein contents of BIP and apoptotic marker Caspase‐3 in primary HUVECs were also significantly decreased by SIRT6 (Figure 3D–H). Therefore, the data suggested that SIRT6 could autonomously modulate H_2_O_2_‐induced ER stress in ECs.

**FIGURE 3:**
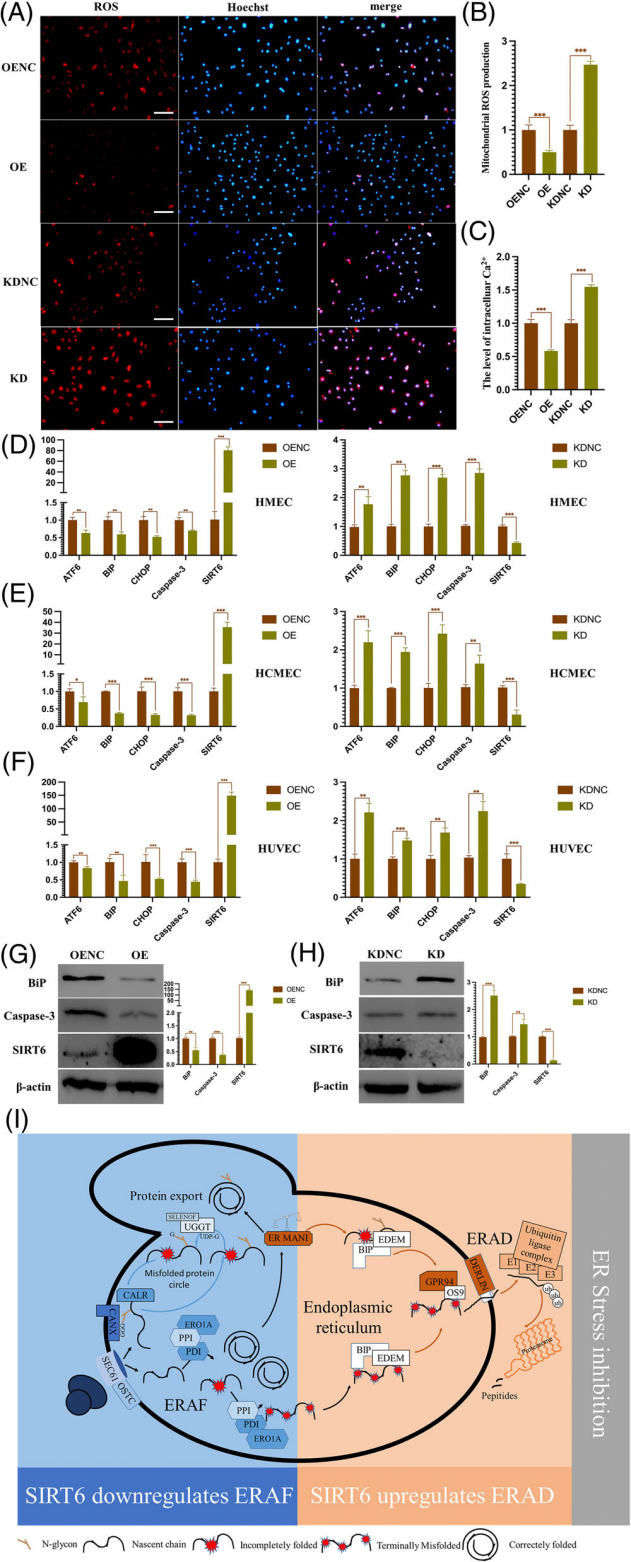
SIRT6 reduced cellular reactive oxygen species (ROS) accumulation and endoplasmic reticulum (ER) stress under oxidative stress. Primary HUVECs were exposed to H_2_O_2_ (400 µM) for 4 h. (A) Assay of ROS levels in HUVECs after H_2_O_2_ exposure. Immunofluorescence for ROS (red) and Hoechst (blue), merged figures for both images. All scale bars: 100 µm. (B) Quantification of values displayed are mean ± standard deviation from five images. (C) Cellular levels of calcium ion were determined in HUVECs after H_2_O_2_ exposure. (D–F) The mRNA levels of ER stress markers and apoptosis marker in HMECs (D), HCMECs (E) and HUVECs (F) after H_2_O_2_ exposure. Western blotting results of BIP and caspase‐3 in primary HUVECs with SIRT6 overexpression (G) or knockdown (H) after H_2_O_2_ exposure. (I) Proposed mechanism of SIRT6 maintaining ER homeostasis through downregulating ERAF and ERAD. The proteins with N‐X‐S/T consensus N‐glycosylation sites are bound by CANX and CALR to assist their folding, and the misfolded proteins are either treated by UGGT, which is activated by SSENLENOF, and recombined with CANX and CALR for misfolded protein cycle, or by MAN1A to end the folding process directly. For proteins without N‐X‐S/T consensus N‐glycosylation sites, they are bound by PDI and PPI that aids their folding. Misfolded proteins are sent by EDEM and BIP to ubiquitin‐mediated degradation. They in turn bind to ubiquitin‐binding enzyme E1, E2, E3 through GPR94, Os9, DERLIN and finally are degraded into peptides by proteasome. Blue‐labelled proteins are downregulated by SIRT6, and red‐labelled proteins are upregulated by SIRT6. SIRT6 downregulated ERAF by downregulating the key proteins SEC61, PDIA1 and ERO1a. SIRT6 upregulated ERAD through upregulating the key protein MAN1 and ubiquitin‐binding enzymes E1, E2, E3. SEC61: protein transport protein; OSTC: oligosaccharyltransferase complex subunit; PDI: protein disulfide isomerase; PPI: peptidyl‐prolyl cis‐trans isomerase; CNX: calnexin; CALR: calreticulin; SELENOF: selenoprotein F; UGGT: UDP‐glucose glycoprotein glucosyltransferase; ERMAN1: endoplasmic reticulum mannosyl‐oligosaccharide 1,2‐alpha‐mannosidase; BIP: endoplasmic reticulum chaperone BiP; Derlin: degradation in the endoplasmic reticulum protein; EDEM: ER degradation‐enhancing alpha‐mannosidase‐like protein; GRP94: heat shock protein 90 beta family member 1; OS9: OS9 endoplasmic reticulum lectin

Collectively, this study showed that SIRT6 could alleviate ER stress by maintaining protein homeostasis in ER (Figure 3I). The impairment of protein processing results in misfolded protein overload in ER, and unfolded proteins response (UPR) was activated to alleviate the stress condition.^4^ Mechanisms of adaptive UPR include reducing the load of customer proteins entering the ER, enhancing the protein folding ability of ER and promoting the ERAD to remove misfolded proteins.^5^, ^6^ In the study, SIRT6 negatively regulated the expression of PDIA1, SEC61a and SEC11a to reduce the proteins translocation to the ER, and upregulated the expression of MAN1A2, UBE2v and UBE2s to enhance the ERAD. In addition, PDI, PPI, CALR and CANX were downregulated by SIRT6, which might reduce the redundant circulation of misfolded proteins in ER (Figure 2F–H). Excessive protein load and ER stress will contribute to ECs’ dysfunctions and the development of cardiovascular diseases (CVDs).^7^ Therefore, SIRT6 would be a novel potential target for maintaining ECs’ functions and preventing some CVDs.

## CONFLICT OF INTEREST

The authors declare that there is no conflict of interest.

## Supporting information

Supporting Information

Supporting Information

Supporting Information

Supporting Information

Supporting Information

Supporting Information

Supporting Information

Supporting Information

Supporting Information
