# Supplementary material for: Multi‐omics analysis reveals the regulation of SIRT6 on protein processing of endoplasmic reticulum to alleviate oxidative stress in endothelial cells
Source: Clin Transl Med. 2022 Aug 29;12(8):e1039. doi: 10.1002/ctm2.1039 (PMC9423132; doi:10.1002/ctm2.1039)
Supplement: Supplementary file 1 — Supporting Information [file CTM2-12-e1039-s009.docx]

**Materials and Methods**

**Cell culture**

Human microvascular endothelial cells (HMECs), human cerebral microvascular endothelial cells (HCMEC) and primary human umbilical endothelial cells (HUVECs) were purchased from Zhongqiaoxinzhou Biotechnology (Shanghai, China). HMECs and HCMECs were cultured in MCBD-131 medium (Sigma–Aldrich, USA) containing 10% FBS (Biological Industries, Israel), EGF (Shanghai Zhongqiaoxinzhou Biotechnology, China), hydrocortisone (Master of Bioactive Molecules, USA) and antibiotics, namely streptomycin and penicillin (Thermo Fisher Scientific, USA) at 37 °C incubator with 5% CO_2_. HUVECs were cultured in endothelial cell medium (Sciencell, USA) added 10% FBS at 37 °C with 5% CO_2_.

Lentivirus and adenovirus vectors either encoding SIRT6, shSIRT6 or empty lentiviral vectors were constructed by Hanbio Biotechnology (Shanghai, China). The sequence of shSIRT6 was 5'-GCTACGTTGACGAGGTCATGA-3'. Lentivirus vectors were individually transfected into HMECs and HCMECs, and adenovirus were transfected into HUVECs. After 12h, the medium of HMECs and HCMECs was replaced and supplemented with 1μg/ml puromycin at 48 h post-transfection for selection; the HUVECs were incubated in a new complete growth medium until the next treatment. The transfection efficiency was validated using western blot and qRT-PCR assays.

**Sample collection**

Discard the culture medium and put the petri dish upside down on the absorbent paper to dry. Add PBS precooled at 4 ℃, gently shake the dish, and then discard PBS. Repeat the above operation twice. Put the petri dish on the ice, add PBS precooled at 4 ℃ to the petri dish, scrape the cells of the petri dish with a clean cell scraper (move quickly), and tilt the petri dish on the ice to make the buffer flow to one side. Use the pipette to transfer the buffer to the precooled centrifugal tube, centrifuge, discard the supernatant, and collect the samples. Liquid nitrogen was used to frozen Samples for 5 minutes and then stored samples at -80℃. For metabolomics, we collected 5 kinds of cells (HMEC-1 WT, SIRT6-kd, SIRT6-kdnc, SIRT6-oe and SIRT6-oenc) and each kind has 6 replicates. For proteomics each kind has 3 replicates. For transcriptomes each kind has 3 replicates.

**RNA sequencing analysis**

## Total RNA was extracted from sample cells by Trizol ®reagents (Invitrogen). The genomic DNA was digested by DNase I (Takara). The RNA mass of sample was determined using 2100 biological analyzer (Agilent), and the RNA quantity was assayed by ND-2000 (NanoDrop Technologies). High quality RNA samples were used for subsequent database construction. A double-terminal library was constructed using ABclonal mRNA-seq-Lib preparation kit (ABclonal, China), and mRNA was purified from total RNA by oligo magnetic beads and cleaved with divalent cations in the first chain synthesis buffer of ABclonal. Then, the first strand cDNA was synthesized using mRNA, random hexamer primers and reverse transcriptase (RNAseH), the second strand cDNA was synthesized using DNA polymerase I, RNAseH and dNTPs. The cDNA fragments were acidified by polyadenylate and ligated to prepare a double-terminal library. cDNA was purified by AMPure XP (Beckman Coulter, USA) and amplified by PCR. PCR amplification was performed with adapter-linked cDNA and adapter primers. Finally, sequencing was performed by Illumina Novaseq 6000. The Clean Reads was sequenced with the designated genome by HISAT2 software to obtain the location information of reference genome. The FPKM value (expected number of Fragments Per Kilobase of transcript sequence perMillions base pairs sequenced) of each gene expression in each sample was calculated by featureCounts software. Then, the differential expression of genes was analyzed by Deseq2 software.

**Proteomic analysis**

SDT (4% (w/w) SDS, 100mM Tris/HCl pH7.6, 0.1m DTT) was used to extract protein of cell samples, extracted proteins were quantified by BCA method. Proteins from each sample were digested by trypsin based on filter aided proteome preparation (FASP) method. The peptides were desalted, freeze-dried and re-dissolved with 40 ul 0.1% formic acid solution, and then quantified by OD280. Each sample was separated by HPLC liquid phase system (Easy nLC). Buffer A is 0.1% formic acid water, and buffer B is 0.1% formic acid acetonitrile (acetonitrile is 84%). The chromatographic column was in liquid equilibrium with 95% buffer A, and the sample was sampled by an automatic injector to the sample feeding column (Thermo Scientific Acclaim PepMap100, 100μm*2cm nanoViper C18), and separated by an analytical column (Thermo scientific EASY column, 10cm, ID75μm, 3μm, C18-A2) with a flow rate of 300 nL/min. After HPLC separation, the MS analysis was performed by Q-Exactive mass spectrometer. The detection was in positive ion mode, the mass range was 300-1800 m/z, the resolution of MS1 was 70000 at 200 m/z, automatic gain control (AGC) target was 1e6, the maximum IT was 50ms, and the dynamic exclusion time was 60.0s. The mass-to-charge ratio of peptides and peptide fragments was collected by following method: after each full scan, 20 fragments (MS2 scan) were collected, the MS2 Activation Type was HCD, Isolation window was 2 m/z, the resolution of MS2 was 17500 at 200 m/z, the Normalized Collision Energy was 30eV and the underfill was 0.1%. The original data of MS analysis were identified and quantified by MaxQuant software.

**Metabolome analysis**

Metabolites were extracted by adding cooled methanol-acetonitrile solution (1:1, v/v) to cell lysates, cell debris were precipitated at -20℃ for 1h and removed by centrifuged (4°C, 14,000 rcf, 20 min). The supernatants were freeze-dried and preserved at -80 ℃. The samples were separated by Agilent 1290 Infinity LC ultra-high-performance liquid chromatography (UHPLC) HILIC column, flow rate 0.5 mL/min, injection volume 2μL, liquid phase composition A: water, 25mM ammonia, 25mM ammonium acetate; B: acetonitrile; during gradient elution procedure. QC samples were also putted into the sample queue to monitor to evaluate the stability and reliability of the experimental data. The primary and secondary spectra of the samples were collected by ABTripleTOF6600 mass spectrometer. The samples were separated by Agilent 1290 InfinityLC UHPLC, and MS analyzed by TripleTOF6600 (ABSCIEX). The samples were detected by positive and negative ion modes in electrospray ionization (ESI), respectively. The original data in Wiff format are converted into mzXML format by ProteoWizard, and then analyzed by XCMS software. Finally, the detected ions were putatively annotated by KEGG, the metabolites with qualitative names were selected as differential metabolites for PLS-DA analysis.

**Bioinformatics and statistical analysis**

For transcriptome and proteome analysis, metascape was used for Kyoto Encyclopedia of Genome (KEGG) pathway enrichment analysis. Only the conditions of P<0.01, minimum count>3 and enrichment factor > 1.5 were considered to be meaningful. The path selected under each condition was corrected by Benjamini-Hochberg (p<0.05FDR<5%). Metabolic pathway analysis and scatterplot mapping of metabolite enrichment were carried out by MetobAnalyst 5.0, and hypergeometric test was used for pathway analysis. The correlation analysis and enrichment bar chart are plotted on the “bioinformatics” website. The Wayne diagram is produced on the “jvenn” website. The column chart is drawn by prism.

**Cell treatment**

Primary HUVECs and lentivirus-transfected HMECs and HCMECs were cultured in 6-well plates with appropriate cell density. When the cell density reached 50%-60%, primary HUVECs were transiently transfected with SIRT6 adenovirus vectors. After 36 hours, the medium HUVECs, HMECs and HCMECs was replaced with serum-free medium for 12 hours. And then, HUVECs, HMECs and HCMECs were stimulated with H_2_O_2_ (400uM) for 1 hour, then cells were harvested for further experiment.

**RNA** **preparation and quantitative real-time PCR**

Total RNAs were isolated from cells using Trizol^®^ reagent RNAiso Plus (Takara, Japan), and then reverse-transcribed cDNA using Prime Script RT Master Mix (Takara, Japan). Real-time quantitative PCR was performed using a CFX Connected™ Real-time PCR Detection System (Bio-Rad, USA) with Maxima SYBR Green/Rox qPCR Master Mix (Thermo Fisher Scientific). The data were normalized to β-actin and fold change was calculated by the comparative CT method (2-∆∆Ct). The primers were listed in Table S4.

**Western Blot**

Proteins were lysed from cells by RIPA buffer (P0013, Beyotime, China) added protease inhibitor and the concentration of proteins was quantified using a BCA kit (P0012, Beyotime, China). Protein lysates were separated by SDS-PAGE and probed with the following primary antibodies: SIRT6 (ab62739, abcam), BiP (ab21685, abcam), Caspase-3 (ab32351, abcam), β-actin (ab8226, abcam). Blots were visualized by the enhanced chemiluminescence (ECL) detection reagents (P0018S, Beyotime, China). The gray band density values were scanned using the iBright CL1500 Imaging System (Thermo Fisher Scientific, USA) and quantified using Image J program.

**Cellular ROS detection**

According to the instructions, MitoSOX™ reagent (M36008, Thermo Fisher Scientific, USA) dissolved in DMSO was diluted in serum-free medium to make 5uM working solution. HUVECs were washed with PBS buffer and covered by MitoSOX™ reagent working solution for 10 minutes at 37˚C in dark. Subsequently, cells were washed with PBS buffer and viewed by epifluorescence microscopy.

**Measurement of calcium ion concentration**

For calcium determination, HUVECs subjected to the aforementioned treatments were collected at sonicated lysed under ultrasound for 10 seconds at a time for 15 cycles. The Calcium Colorimetric Assay kit (Elabscience, China) was used to detect the intracellular Ca^2+^ concentration at absorbance λ = 610 nm using a microplate reader (Biorad, USA).

**Statistical analysis**

Obtained data were from three independent replicates The results in the figures were expressed as mean ± standard deviation. unpaired two-tailed Student’s t-test was used for comparisons between different groups. Differences were considered statistically significant if the P-value was smaller than 0.05. *P ≤ 0.05; **P ≤ 0.01; ***P ≤ 0.001; ns, P >0.05, no significance.
