# Supplementary figures and images for: Multi‐omics analysis reveals the regulation of SIRT6 on protein processing of endoplasmic reticulum to alleviate oxidative stress in endothelial cells
Source: Clin Transl Med. 2022 Aug 29;12(8):e1039. doi: 10.1002/ctm2.1039 (PMC9423132; doi:10.1002/ctm2.1039)

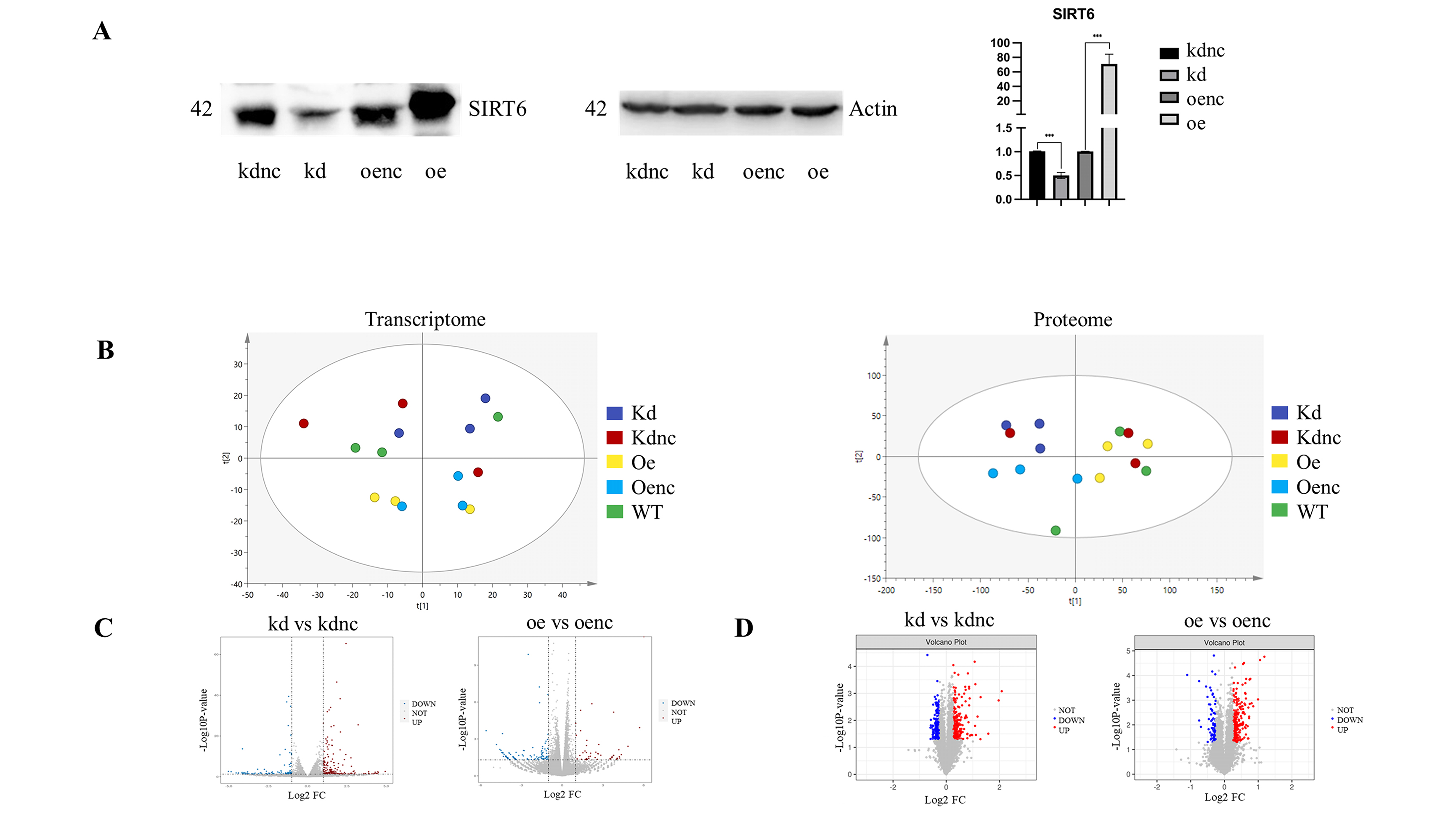

Supplement: Supplementary file 6 — Supporting Information [file CTM2-12-e1039-s008.jpg]

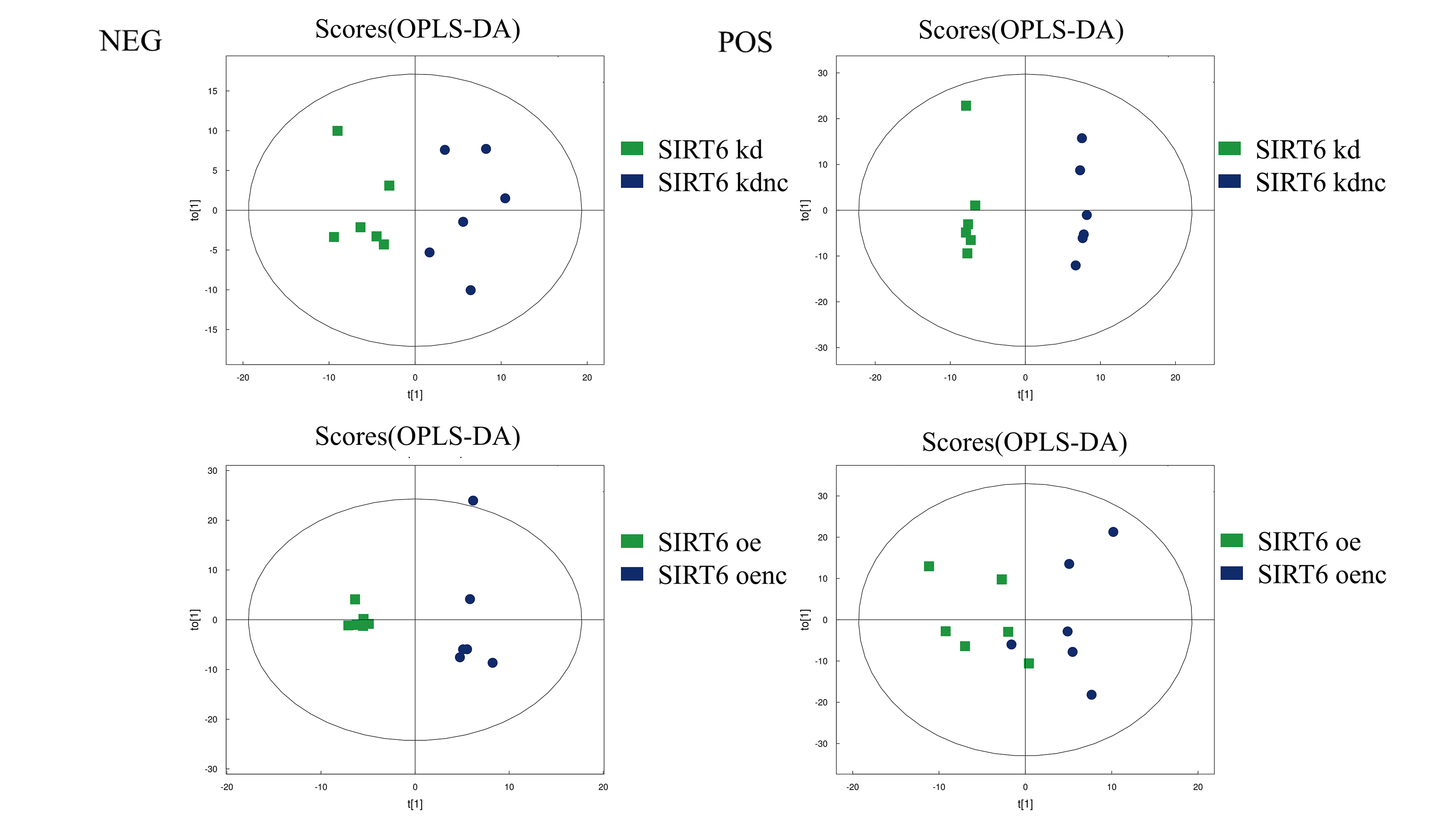

Supplement: Supplementary file 7 — Supporting Information [file CTM2-12-e1039-s005.jpg]

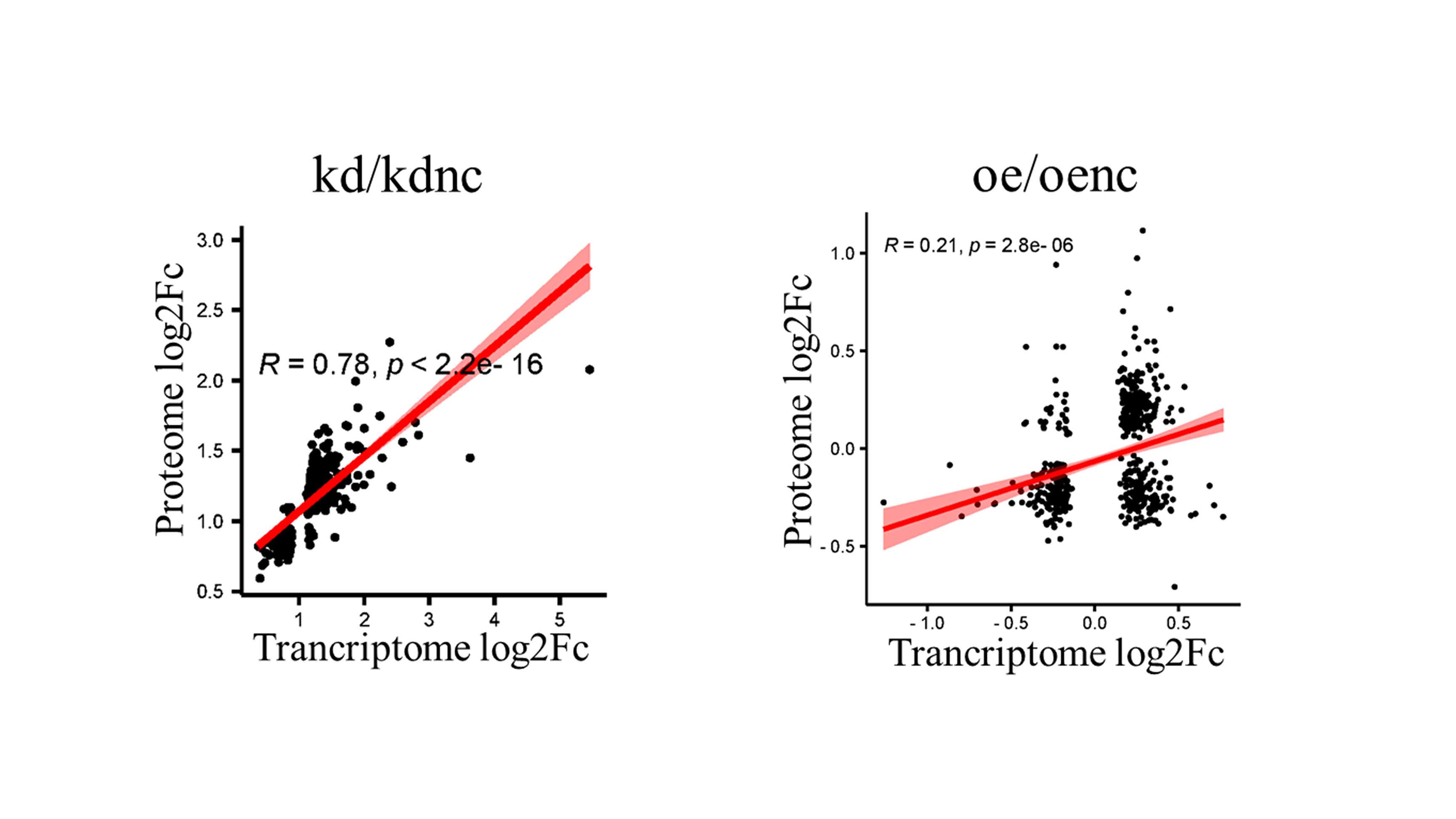

Supplement: Supplementary file 8 — Supporting Information [file CTM2-12-e1039-s001.jpg]

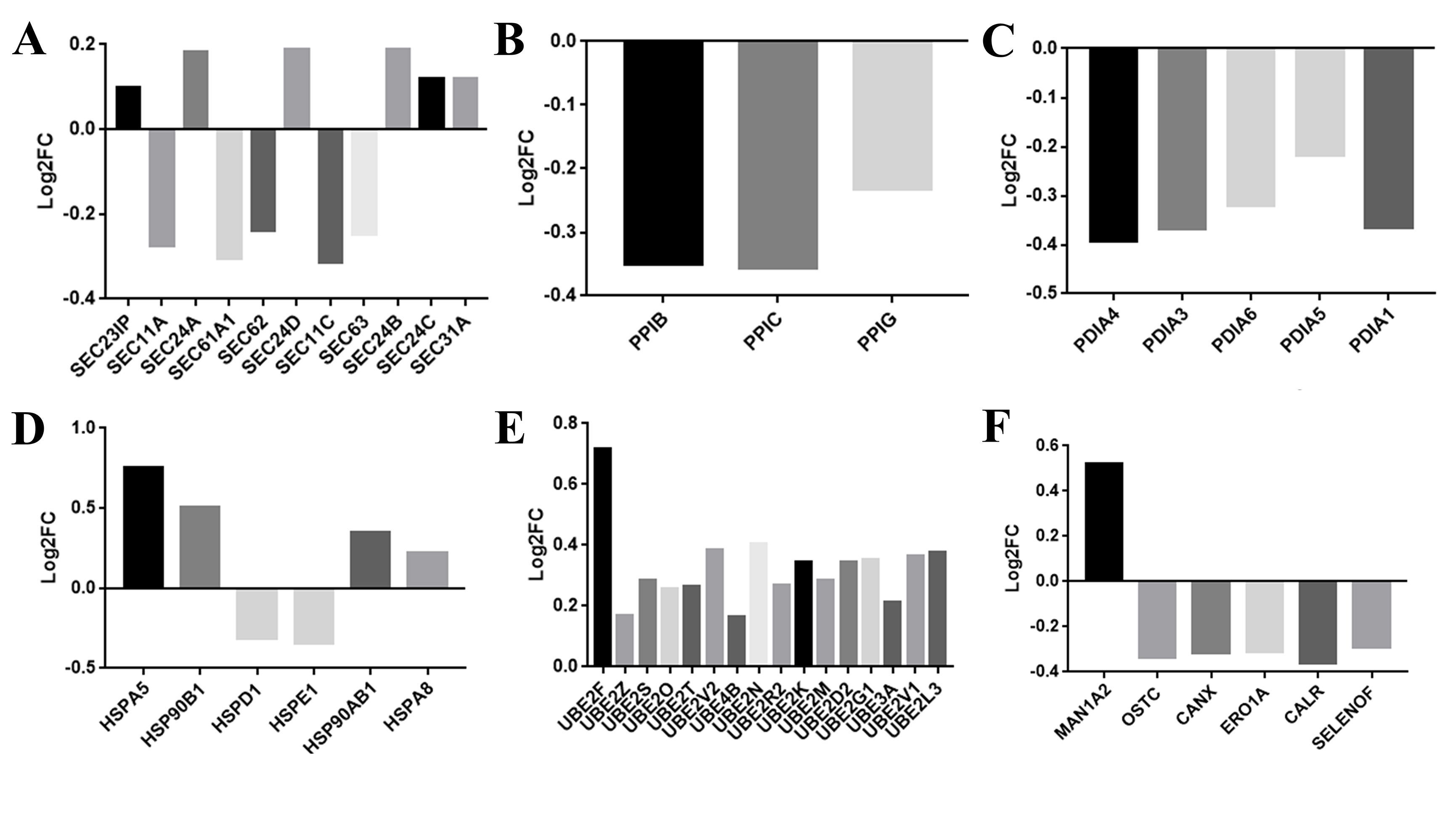

Supplement: Supplementary file 9 — Supporting Information [file CTM2-12-e1039-s002.jpg]
